# Surgical Treatment for Esophageal Leiomyoma: 13 Years of Experience in a High-Volume Tertiary Hospital

**DOI:** 10.3389/fonc.2022.876277

**Published:** 2022-04-11

**Authors:** Gu-Ha A-Lai, Jian-Rong Hu, Peng Yao, Yi-Dan Lin

**Affiliations:** ^1^ Department of Thoracic Surgery, West China Hospital, Sichuan University, Chengdu, China; ^2^ Operating Room of Anesthesia Surgery Center, West China Hospital/West China School of Nursing, Chengdu, China

**Keywords:** esophagus, leiomyoma, enucleation, thoracotomy, thoracoscopy

## Abstract

**Background:**

Esophageal leiomyoma is the most common benign tumor in the esophagus. Thoracotomy and thoracoscopy are both elective for esophageal leiomyoma enucleation. This study aimed at presenting surgical experience in our center and exploring more suitable surgical methods for different situations.

**Methods:**

We conducted this retrospective study by collecting data from patients who underwent esophageal leiomyoma enucleation through thoracotomy or thoracoscopy from January 2009 to November 2021 at West China Hospital Sichuan University.

**Results:**

A total of 34 patients were enrolled for analysis. All patients were diagnosed with a single esophageal leiomyoma. There were 25 men and 9 women. The mean age was 44.41 years (range, 18–72 years), the mean longest diameter was 4.99 cm (range, 1.4–10 cm), and the esophagus was thoroughly circled with leiomyoma in 10 patients, 10 patients underwent thoracotomy to enucleate leiomyoma, while others underwent thoracoscopic enucleation. No perioperative deaths occurred. Between the thoracotomy group and thoracoscopy group, baseline characteristics were comparable except for gastric tube status (p = 0.034). Patients were inclined to undergo the left lateral surgery approach (p = 0.001) and suffered esophagus completely encircled by leiomyoma (p = 0.002). Multivariable logistic regression analysis demonstrated that the left lateral surgery approach (p = 0.014) and esophagus completely encircled by leiomyoma (p = 0.042) were risk factors for thoracotomy of leiomyoma enucleation, while a larger tumor size demonstrated no risk. The median follow-up time was 63.5 months, and no deaths or recurrence occurred during the follow-up period.

**Conclusion:**

Thoracotomy enucleation of the leiomyoma was recommended when the esophagus was thoroughly encircled by the leiomyoma and the left lateral surgery approach was needed. However, tumor size demonstrated less value for selecting a surgical approach.

## Background

Leiomyoma is most common in the uterus, esophagus, and small bowel. Esophageal leiomyoma accounts for 70%–80% of esophageal submucosal tumors and less than 1% of all esophageal neoplasms (1, 2). Patients suffer esophageal leiomyoma mainly between 20 and 50 years of age and with a predominance in men. Many esophageal leiomyomas are found accidentally without any symptoms, while dysphagia, epigastric discomfort, and retrosternal pain are relatively more common than other symptoms, and single esophageal leiomyoma is more predominant than multiple leiomyomas, which is often found in the lower two-thirds of the esophagus (2–4).

It is known that esophageal leiomyoma has an extremely low possibility of converting malignancy, and surgical treatment is traditionally recommended for tumors that are symptomatic or larger than 5 cm or with unclear biological behavior, while observation is conducted for small tumors (4, 5). Thoracoscopic surgery has developed rapidly since Everitt reported the first thoracoscopic approach (6). Thoracoscopy and thoracotomy subsequently became elective choices for esophageal leiomyoma enucleation, while the thoracoscopic approach could decrease incision size, postoperative trauma, hospital stay duration, and postoperative pain (7–9). Although the thoracoscopic approach provides many advantages over thoracotomy, it cannot be performed under some circumstances that may increase surgical difficulty and risk, such as giant leiomyoma, severe thoracic adhesion, and other special statuses. Hence, thoracotomy remains an indispensable and elective choice for esophageal leiomyoma enucleation. Therefore, this study aimed at demonstrating surgical treatment experience in our center and exploring how to choose a suitable surgical approach for esophageal leiomyoma enucleation under different circumstances.

## Methods

### Patients

A total of 34 patients who underwent surgical esophageal leiomyoma enucleation from January 2009 to November 2021 at the Department of Thoracic Surgery, West China Hospital Sichuan University, were retrospectively included in this study. Surgical approaches included a minimally invasive approach and a thoracotomy approach. This retrospective study was approved by the Ethics Committee of West China Hospital of Sichuan University, and the requirement for informed consent was waived.

### Preoperative Assessment

All patients underwent routine examination of chest computed tomography (CT) scans, esophagography, esophagogastroscopy, elective endoscopic ultrasonography, routine blood tests, electrocardiograms, and routine medical inquiry before admission to the hospital. All the above could ensure a proper surgical indication and exclude patients who could not endure surgery. Leiomyoma thoroughly encircling the esophagus was defined as leiomyoma tissue that can be found all around the esophagus circumferentially at one cross section of the CT scan.

### Surgical Approach

Thoracoscopic surgery had been developed, and video-assisted thoracoscopic surgery had been the first choice for all suitable patients in our center earlier than 2009. With the rapid development of robot-assisted thoracoscopic surgery in recent years, this mature technique has also been masterly conducted in our center with abundant experience. All the patients in this study received the above preoperative assessment by their chief doctor before admission, and the patients were subsequently discussed by all doctors in our department to determine whether they featured proper surgical indications and specific surgical approaches after admission. All patients suitable for the thoracoscopy approach underwent VATS leiomyoma enucleation, except for some cases, such as giant tumors, complicated tumor shapes, and other special statuses that require thoracotomy. Concerning the approach of surgical incision, it was selected mainly based on the main side affected by the tumor considering the specific tumor location at the same time; according to the anatomy features of the esophagus location and impact of the adjacent organs, the approach *via* the left side was preferred if the tumor is located in the esophagogastric junction, and the right-side approach was often chosen if the tumor is located in the lower esophagus without reaching the esophagogastric junction. All these patients underwent transthoracic surgery under general anesthesia with intraoperative single-lung ventilation, and the lateral decubitus position was used.

After completion of all the procedures before initial enucleation, the first step was to localize the tumor location and incise the mediastinal pleura longitudinally. The second step was to conduct longitudinal myotomy and gradually dissect the leiomyoma. We could suture the tumor with a string to drag the tumor for convenient dissection from different directions. The third step was to check the integrity of the esophageal mucosa after complete enucleation. Mucosa repair was performed once mucosa rupture was found. Finally, continuous or interrupted sutures were performed for the muscular layer.

### Follow-Up

Patients underwent outpatient or telephone follow-up every 3 months in the first year after surgery and once a year for the next years. Patients received telephone follow-ups if regular outpatient visits were unavailable. Follow-up was conducted up to November 2021 or the date of death. Recurrence-free time was measured from the date of operation to the date of recurrence. Three patients were lost to follow-up, and the mean follow-up interval was 68 months.

### Statistical Analysis

Statistical analysis was performed using SPSS 26.0 software (SPSS Corp., Chicago, IL, USA). Continuous variables were demonstrated by the mean ± standard deviation or range, the median, and the interquartile range for abnormal distribution; data were analyzed with Student’s t-test or the Mann–Whitney U test in case of abnormal distribution. For categorical data, the chi-square or Fisher’s exact test was applied. In addition, binary logistic regression analysis was performed for risk factor analysis, and variables with a p value ≤0.10 in the baseline characteristic analysis or that were thought to be significant clinically were included. A p value less than 0.05 in the two-tailed test was considered significant.

## Results

### Patient Characteristics

A total of 39 patients were extracted from our medical records: 5 patients underwent endoscopic leiomyoma enucleation after careful preoperative evaluation, and the remaining 34 patients underwent surgical leiomyoma enucleation and were enrolled in this study. Patient characteristics are shown in **Table 1**. There were 25 men and 9 women with a mean age of 44.41 years. All the patients featured a single leiomyoma, and no patients received preoperative biopsy. Most cases were asymptomatic and located in the middle and lower thirds of the esophagus. The mean longest tumor size reached 4.99 cm, and most patients also featured no calcification. Twenty-four patients underwent minimally invasive thoracoscopic surgery, while 10 patients underwent a thoracotomy approach, including 1 conversion patient. Nine patients underwent the left approach, while 25 patients underwent the right approach, and a total of 3 patients suffered intraoperative mucosa rupture. Concerning postoperative characteristics, only 1 patient from the thoracoscopic surgery group suffered esophageal leakage, who was diagnosed with a thoroughly circling tumor around the esophagus, and the patient received a second operation for mucosa repair on postoperative day 3. The was cost more than 230,000 RMB while the postoperative hospital duration was nearly 100 days, which demonstrated an extreme value compared to the others, so the data shown below excluded the patient. The mean hospital cost reached 31,572.88 RMB, and the mean postoperative hospital duration and postoperative oral intake time were 6.06 and 3.97 days, respectively. In addition, gastric tube duration, postoperative drainage tube duration, and drainage volume reached 2.73 days, 3.73 days, and 521 ml, respectively. No perioperative deaths occurred among the patients.

**Table 1:** Baseline characteristics of all esophageal Leiomyoma patients.

Variables	Mean [range]/n
Age	44.41 [18–72]
Sex	
Male	28
Female	9
Preoperative biopsy	0
Tumor location	
Upper	6
Middle	15
Lower	13
Symptom	
Yes	19
No	15
Significant pleura adhesion	
Yes	2
No	32
Comorbidity	
Yes	9
No	25
Tumor number	
Single	34
Multiple	0
Largest tumor size	4.99 [1.4–10]
Body mass index	23.34 [18.40–27.06]
Tumor shape	
Thorough encircling esophagus	10
Partial encircling esophagus	24
Calcification	
Yes	6
No	28
Surgery method	
Thoracotomy	10
Thoracoscopy	24
Surgery approach	
Left lateral	9
Right lateral	25
Intraoperative mucosa rupture	
Yes	3
No	31
Complication	
Yes	1
No	33
Perioperative death	
Yes	0
No	34
Gastric tube	
Yes	25
No	9
Cost	31,572.88 [16,063.19–73163.48]
Postoperative hospital duration	6.06 [3–10]
Oral intake time	3.97 [1–7]
Gastric tube duration	2.73 [0–7]
Drainage tube duration	3.73 [1–9]
Drainage volume	521 [80–1,960]
Follow-up death	0
Follow-up recurrence	0
Follow-up interval	68 [3–151]

### Comparison Between the Thoracotomy Group and Thoracoscopy Group

Ten patients completely underwent thoracotomy, and 24 patients successfully completed thoracoscopic surgery of esophageal leiomyoma enucleation. One patient who received second mucosa repair surgery in the thoracoscopy group was excluded from the data analysis of cost, drainage tube duration, drainage volume, postoperative hospital duration, postoperative oral intake duration, and gastric tube duration. Baseline characteristics were comparable in terms of sex, comorbidity, symptom status, calcification status, tumor location, tumor size, postoperative hospital duration, and other characteristics. However, patients in the thoracotomy group were more likely to undergo enucleation *via* the left approach (p = 0.001) and suffer esophagus with thorough tumor encircling (p = 0.002) than those in the thoracoscopy group, as shown in **Table 2**. In addition, the cost in the thoracoscopy group tended to be higher than that in the thoracotomy group, but the difference was not significant (p = 0.068). The thoracotomy group patients also tended to have gastric tubes placed (p = 0.034), as shown in **Table 3**.

**Table 2:** Characteristics of the thoracoscopy and thoracotomy groups.

Characteristics	Thoracoscopy group (n = 24)	Thoracotomy group (n = 10)	p value
Age	44.50 [32.50, 52.25]	44 [33.75, 56]	0.733
Sex			1.00
Female	6	3	
Male	18	7	
Tumor location			0.210
Upper	6	0	
Middle	10	5	
Lower	8	5	
Symptom			0.451
Yes	12	3	
No	12	7	
Body mass index	23.53 ± 3.58	22.88 ± 2.54	0.606
Gastric tube			0.034
Yes	15	10	
No	9	0	
Comorbidity			0.692
Yes	7	2	
No	17	8	
Largest tumor size	4.75 [3.50, 6.00]	5.0 [2.88, 7.63]	0.718
Tumor shape			0.002
Thorough encircling esophagus	3	7	
Partial encircling esophagus	21	3	
Calcification			1.000
Yes	4	2	
No	20	8	
Surgery approach			0.001
Left lateral	2	7	
Right lateral	22	3	
Significant pleura adhesion			0.508
Yes	1	1	
No	23	9	

**Table 3:** Outcomes between the thoracotomy and thoracoscopy groups.

Characteristics	Thoracoscopy group (n = 24)	Thoracotomy group (n = 10)	p value
Intraoperative mucosa rupture			1.000
Yes	2	1	
No	22	9	
Complication			1.000
Yes	1	0	
No	33	10	
Cost	28158.10 [23,103.22, 38,111.53]	24,810.05 [18,330.12, 28,496.50]	0.068
Postoperative hospital duration	6 [5, 7]	6.50 [4.75, 8]	0.365
Oral intake time	4 [3, 4]	5.5 [2.75, 6.25]	0.106
Gastric tube duration	2 [0, 4]	4 [3, 5.25]	0.006
Drainage tube duration	3 [3, 4]	3.5 [2.75, 5.50]	0.638
Drainage volume	420 [260, 730]	430 [290, 887.5]	0.736
Follow-up lost			0.201
Yes	1	2	
No	23	8	
Follow-up interval	54.5 ± 7.69	100.4 ± 12.24	0.003
Follow-up death			
Yes	0	0	
No	24	10	
Follow-up recurrence			
Yes	0	0	
No	24	10	

### Multivariable Analysis of Thoracotomy

We included longest tumor size, surgical approach, and tumor thoroughly encircling status in multivariable logistic regression analysis for thoracotomy. We found that a tumor size greater than 5 cm was not a risk factor for thoracotomy, with an OR of 0.597 (95% Cl, 0.062–5.788, p = 0.656). In contrast, surgery *via* the left approach showed a significant risk factor for thoracotomy with an OR of 15.875 (95% Cl, 1.736–145.165, p = 0.014), while tumors thoroughly encircling the esophagus also demonstrated a risk factor for thoracotomy with an OR of 10.061 (95% Cl, 1.087–93.107, p = 0.042), as shown in **Table 4**.

**Table 4:** Binary logistic regression analysis of the thoracotomy approach.

Variables	OR	95% Cl	p
Tumor shape			0.042
Partial encircling esophagus	1	–	
Thorough encircling esophagus	10.061	1.087–93.107	
Surgery approach			0.014
Right lateral	1	–	
Left lateral	15.875	1.736–145.165	
Tumor size			0.656
≤5 cm	1	–	
>5 cm	0.597	0.062–5.788	

### Follow-Up Outcomes

The follow-up interval varied from 3 to 151 months, and the mean follow-up interval reached 68 months among all 34 patients. A total of 3 patients were lost in the follow-up period, and the loss rate was lower than 10%, which was an acceptable loss rate; 2 were from the thoracotomy group, and 1 was from the thoracoscopy group. The follow-up interval was significantly longer in the thoracotomy group. No death or recurrence occurred in the follow-up period.

## Discussion

This study aimed at exploring the epidemiology, surgical outcomes, and suitable surgical approaches of esophageal leiomyoma patients in our department. We demonstrated the surgical treatment experience of a large single-center case of esophageal leiomyoma. As a local large tertiary center in southwestern China, we annually conducted more than 6,000 thoracic surgeries, but only 34 cases underwent surgery in our center during the last 13 years, which was in accordance with a low overall incidence of 0.006%–0.1% in a previous report. This study demonstrated that leiomyoma has features of significant male, single tumor, lower location and less calcification predominance, which is similar to a worldwide review of 838 cases (2, 3). In addition, most patients featured symptoms, and the most common symptoms were epigastric distress and dysphagia, which was in agreement with a previous study (2). This may be the reason that the mean longest tumor diameter was only 4.99 cm, and many patients underwent surgeries owing to significant symptoms even though the tumor was not large enough. Esophageal leiomyoma was relatively easy to diagnose by CT scan and upper gastrointestinal contrast-enhanced X-ray *via* the significant signs; after considering the diagnosis of esophageal leiomyoma which was a benign disease, patients generally would not be recommended to receive preoperative biopsy by most surgeons, mainly owing to concerns of esophageal leakage, bleeding, and other complications, and indeed no patients underwent preoperative biopsy in this study.

According to experience of surgical difficulty, we first divided the relationship between esophagus and tumor into two statuses: status A was esophagus thoroughly encircled by tumor, and status B was partial esophagus not reached circumferentially; 10 patients were divided into status A, while the remaining 24 patients belonged to status B. Subsequently, we found that status A was indeed more common in the thoracotomy leiomyoma enucleation group and identified as a risk factor for thoracotomy by multivariable logistic analysis. The results confirmed our designation: patients suffering status A had more difficulty performing thoracoscopic surgery and needed thoracotomy surgery. In addition, we also found that surgery *via* the left approach was significantly more common in the thoracotomy group than in the thoracoscopy group and that the left approach was also a risk factor for thoracotomy. However, tumor size was comparable between the two groups. According to the anatomical features of the esophagus, it mainly leans right in the thoracic region and is near the descending aorta on the left side, so the right surgical approach for upper/middle third esophageal leiomyoma could create more operating space and avoid affecting the heart and major vessels. However, it was quite different for the lower third esophagus, especially for the gastroesophageal junction. The lower esophagus is impacted by the heart even though it turns left. Therefore, surgeons may need to select a thoracotomy approach when the main tumor is on the left side to ensure safety for third esophageal leiomyoma, especially for gastroesophageal junction leiomyoma which complicated with a status of thoroughly encircling. Thus, operation could be conducted safely under direct view without severe compact of the heart. However, tumor size was similar between the two groups, and a risk factor for thoracotomy was not identified, even if the tumor size was near 10 cm, which was different from previous recommendations (3, 10–13). Some studies also reported that the thoracoscopy approach was feasible for tumors larger than 5 cm (14, 15).

Among all the patients, only one patient who underwent thoracoscopic leiomyoma enucleation suffered postoperative complications of esophageal leakage. This was a 35-year-old male patient who was diagnosed with esophageal leiomyoma at the lower esophagus that reached the esophagogastric junction. The longest tumor size reached 6 cm and thoroughly encircled the esophagus. The patient suffered dyspnea, and drainage became purulent on postoperative day 2. Then, endoscopy examination showed two small esophageal leakages on postoperative day 3. Subsequently, he received reoperation to clear the acute pyothorax of both sides and to repair the leakage. Following a long duration of conservative treatment, the patient was successfully discharged. Reviewing this case, we believe that the most suitable and safe surgery should be thoracotomy enucleation under special circumstances in which the esophagus is encircled thoroughly by the tumor. This was in agreement with our conclusion. In addition, 3 patients were found to have mucosa rupture intraoperatively with a water test and received repair at once. All patients recovered successfully, and all were discharged within 9 postoperative days. According to our experience, the thoracic surgical approach was still more appropriate for lower third esophageal leiomyoma which was completely located in the thoracic junction, while leiomyoma was located in the gastroesophageal junction, especially for leiomyoma extending into abdominal cavity; the laparoscopy or laparotomy transhiatal approach should be considered seriously which could avoid chest incision.

At present, standard guidelines for esophageal leiomyoma are scarce. Observation, endoscopic surgery, thoracoscopy surgery, and thoracotomy surgery are all options (7). Some scholars recommended symptomatic tumors, increased tumor size, and uncertain biological behavior as surgical indications (7, 9). Asymptomatic tumors smaller than 5 cm were suggested for observation and follow-up by these scholars (16, 17). However, another scholar thought that 1–5-cm leiomyoma should also be excised once found regardless of whether it was symptomatic, so we could confirm the histology and avoid any possibility of malignant degeneration. Leiomyoma enucleation by thoracotomy is the most traditional and common approach (7, 18), and all kinds of leiomyoma enucleation can be completed *via* this approach. Thoracoscopic surgery also developed well and has been proven effective and safe with wide performance, especially for tumor sizes between 1 and 5 cm (9, 11, 19–21). Other studies viewed that tumors greater than 5 cm were also not a contradiction for thoracoscopic surgery, which was similar to our study (22, 23). With the development of endoscopy techniques, leiomyoma resection *via* endoscopic submucosal dissection (ESD) and endoscopic submucosal tunnel dissection (ESTD) has also been proven feasible and safe for limited patients (24–27). However, endoscopic resection of leiomyoma could only be a complementary approach to surgical resection owing to limitations which include bleeding, perforation, infection, incomplete resection for large tumor, and other related complications. Besides, urgent measures were limited for ESD and ESTD once severe complications occurred. With the fast development of robot-assisted thoracoscopic surgery (RATS), more and more difficult surgeries can be conducted with the advantages of robots including a 3-dimensional surgical view and extremely flexible robot hands. Studies also verified the advantages of RATS for esophageal leiomyoma (28, 29). RATS application could be explored further in the future.

No deaths or reoccurrences occurred in our study during either the perioperative time or the follow-up period. Previous studies were also in agreement with our results (3, 13, 14, 21, 30). There was also no malignant transformation found in our study, which was also consistent with most previous results, while a comprehensive study concerning more than 800 patients worldwide reported that 2 cases transformed from leiomyoma to leiomyosarcoma (31). At present, leiomyoma is a benign tumor and is usually thought to feature low malignant transformation and the possibility of recurrence, which can be thoroughly cured once resected. On the other hand, there was no malignant transformation, death, or recurrence with a mean follow-up interval of 68 months in our study, also confirming the view again.

This study was accompanied by several limitations. First, a sample of 34 cases was relatively small, although the sample even exceeded the vast majority of studies of esophageal leiomyoma. In addition, some parameters, such as operative time and intraoperative bleeding volume, were partially missing and could not be analyzed. Moreover, several surgical teams may have different surgical approach preferences even though they are all experienced in thoracic surgery. Finally, this study included only Chinese patients, and the results could not be expanded to other races.

## Conclusion

Thoracotomy and thoracoscopy enucleation of esophageal leiomyoma were both effective and safe, and a thoracoscopy approach should be the first choice owing to its minimally invasive nature; however, thoracotomy surgery should be recommended when the tumor thoroughly circles the esophagus and the left approach is needed. Tumor size was not a decisive factor for thoracotomy leiomyoma enucleation. Multicenter and large sample studies are urgently needed to help choose the proper surgical approach in the future.

## Data Availability Statement

The data analyzed in this study is subject to the following licenses/restrictions: all the data were available for appropriate request from responding author. Requests to access these datasets should be directed to linyidan.academy@foxmail.com.

## Ethics Statement

The studies involving human participants were reviewed and approved by the Ethics Committee of West China Hospital of Sichuan University. Written informed consent for participation was not required for this study in accordance with the national legislation and the institutional requirements.

## Author Contributions

Conception and design: Y-DL, G-HA-L. Administrative support: Y-DL. Provision of study materials or patients: J-RH, PY. Collection and assembly of data: G-HA-L, JR Hu. Data analysis and interpretation: G-HA-L, PY. Manuscript writing: all authors. All authors contributed to the article and approved the submitted version.

## Funding

This work was supported by the National Natural Science Foundation of China (No. 81672291) (to Y-DL).

## Conflict of Interest

The authors declare that the research was conducted in the absence of any commercial or financial relationships that could be construed as a potential conflict of interest.

## Publisher’s Note

All claims expressed in this article are solely those of the authors and do not necessarily represent those of their affiliated organizations, or those of the publisher, the editors and the reviewers. Any product that may be evaluated in this article, or claim that may be made by its manufacturer, is not guaranteed or endorsed by the publisher.
